# Hexamethylentetramin: MAK-Begründung, Nachtrag

**DOI:** 10.34865/mb10097kskd11_1ad

**Published:** 2026-03-30

**Authors:** Andrea Hartwig, MAK Commission

**Affiliations:** 1 Institut für Angewandte Biowissenschaften, Abteilung Lebensmittelchemie und Toxikologie, Karlsruher Institut für Technologie (KIT) Adenauerring 20a, Geb. 50.41 76131 Karlsruhe Deutschland; 2 Ständige Senatskommission zur Prüfung gesundheitsschädlicher Arbeitsstoffe, Deutsche Forschungsgemeinschaft, Kennedyallee 40, 53175 Bonn, Deutschland. Weitere Informationen: Ständige Senatskommission zur Prüfung gesundheitsschädlicher Arbeitsstoffe | DFG

**Keywords:** Hexamethylentetramin, Sensibilisierung, Formaldehydabspalter, Reizwirkung, MAK-Wert, Maximale Arbeitsplatzkonzentration, Spitzenbegrenzung, Mutagenität, Luft, air

## Abstract

The German Senate Commission for the Investigation of Health Hazards of Chemical Compounds in the Work Area (MAK Commission) summarized and re-evaluated the data for hexamethylenetetramine [100-97-0] to derive an occupational exposure limit value (maximum concentration at the workplace, MAK value) considering all toxicological end points. Relevant studies were identified from a literature search. Clinical findings demonstrate that frequent or regular contact with hexamethylenetetramine can lead to skin sensitization. This was confirmed by a study in animals. Therefore, the “Sh” designation has been retained. There are no data for respiratory sensitization. Hexamethylenetetramine releases formaldehyde in aqueous solution only in very small amounts at physiological pH levels. Therefore, a carcinogenic effect on the nasal mucosa epithelium, as with formaldehyde, is not expected. It is unlikely that the formaldehyde released from hexamethylenetetramine reaches the germ cells in active form. Oral administration in rats and mice of up to 2 g/kg body weight (bw) for up to 104 weeks did not result in systemic toxicity. The histopathological examination of male and female rats did not reveal any effects after administration of a hexamethylenetetramine dose of 50 mg/kg bw in the drinking water for 50 weeks. This NOAEL was used to derive a MAK value of 20 mg/m^3^ for the inhalable fraction of hexamethylenetetramine. As the critical effect is systemic, Peak Limitation Category II has been set with an excursion factor 8. There are no reliable data for prenatal developmental toxicity and hexamethylenetetramine has therefore been assigned to Pregnancy Risk Group D. Skin contact is not expected to contribute significantly to systemic toxicity.

**Table:** 

**MAK-Wert (2025)**	**20 mg/m^3^ E ≙ 3,4 ml/m^3^ (ppm)**
**Spitzenbegrenzung (2025)**	**Kategorie II, Überschreitungsfaktor 8**
	
**Hautresorption**	**–**
**Sensibilisierende Wirkung (1997)**	**Sh**
**Krebserzeugende Wirkung**	**–**
**Fruchtschädigende Wirkung (2025)**	**Gruppe D**
**Keimzellmutagene Wirkung**	**–**
	
**BAT-Wert **	**–**
	
Synonyma	Hexamin Methenamin Urotropin
Chemische Bezeichnung (IUPAC-Name)	1,3,5,7-Tetraazatricyclo[3.3.1.1^3,7^]decan
CAS-Nr.	100-97-0
Formel	Strukturformel von Hexamethylentetramin. 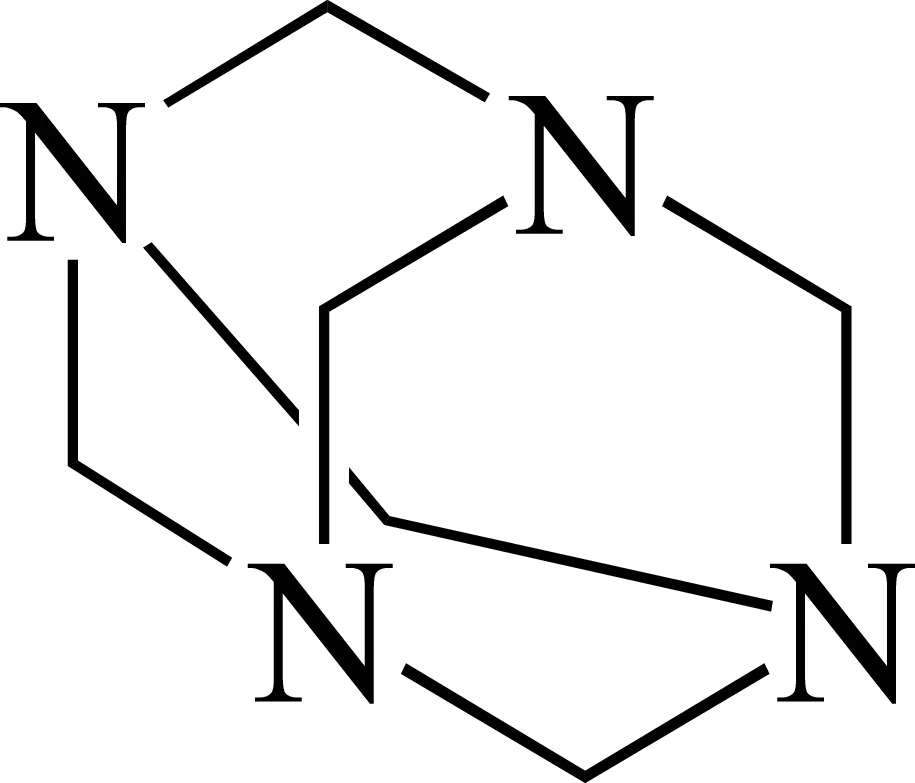
	C_6_H_12_N_4_
Molmasse	140,19 g/mol
Dichte bei 22 °C	1,331 g/cm^3^ (Feststoff) (Dreyfors et al. 1989)
Dampfdruck bei 20 °C	0,0013 hPa (ECHA 2024) 0,0005 hPa (Klipping und Stranski 1958 in ECHA 2024)
Dampfsättigung bei 20 °C	1,3 ml/m^3^ (7,5 mg/m^3^) aus Dampfdruck berechnet
log K_OW_	–2,18 (exp., pH-Wert 7–9) (ECHA 2024)
pH	8,4 bei 28 g/l (NCBI 2025)
pKs-Wert bei 20 °C	8,4 (ECHA 2024) 8,85 (RÖMPP Team und Schwab 2019)
Löslichkeit	895 g/l Wasser bei 20 °C (ECHA 2024) 874 g/l Wasser bei 40 °C (ECHA 2024)
**1 ml/m^3^ (ppm) ≙ 5,817 mg/m^3^**	**1 mg/m^3^ ≙ 0,172 ml/m^3^ (ppm)**
	
Hydrolysestabilität	Stabil bei pH 7,4 (Strom und Jun 1980; Abschnitt 3). Aus einem Molekül Hexamethylentetramin können sechs Moleküle Formaldehyd und vier Moleküle Ammoniak entstehen (Abschnitt 3).
Verunreinigungen	– (techn. Produkt Reinheit > 99 %, ACGIH 2020)
Einsatzverbote	Einsatz in der EU in Lebensmitteln (EFSA ANS Panel 2014) und als Biozid verboten (ECHA 2025 a)
Einsatzkonzentration und Verwendung	0,15 % in Kosmetika (Europäisches Parlament und Europäischer Rat 2022), < 1 % in Waschlösungen (Citrus) (US EPA 2006), 2–4 g/Tag therapeutische Dosis bei Zystitis (57 mg/kg KG und Tag) (SCHER 2007), < 1 % als Korrosions-Inhibitor (NICNAS 2013), 600 mg/kg Zusatzstoff für den Silierprozess von Futtermitteln (EFSA FEEDAP Panel 2015), zum Aushärten phenolischer Harze, Vulkanisierungsbeschleuniger, Biozid in Bearbeitungs- und Schneideflüssigkeiten (k. w. A.; BAuA 2008)

Hinweis: Formaldehydabspalter. Der Stoff kann gleichzeitig als Dampf und Aerosol vorliegen.

Zu Hexamethylentetramin liegen eine Begründung (Henschler 1991) und ein Nachtrag (Greim 1997) zur sensibilisierenden Wirkung vor. Es erfolgt eine Neubewertung der Daten zur Formaldehydabspaltung nach aktuellem Vorgehen der Kommission (DFG 2025).

Aufgrund der Zunahme an Harnwegsinfektionen mit antibiotikaresistenten Bakterien wird aktuell eine erneute therapeutische Verwendung von Hexamethylentetramin diskutiert (Chatterjee et al. 2025; Davidson et al. 2024; Harding et al. 2022; Li et al. 2024).

Auch die Möglichkeit einer therapeutischen Anwendung in der Behandlung von Tumoren wird aufgezeigt (Altinoz et al. 2019).

## 1 Allgemeiner Wirkungscharakter

Hexamethylentetramin wirkt beim Menschen und am Tier sensibilisierend an der Haut.

Hexamethylentetramin zeigt eine sehr geringe Reizwirkung nach okklusiver Auftragung an der Kaninchenhaut, ist jedoch nicht augenreizend.

Orale Gaben von 8 g Hexamethylentetramin/Tag an Patientinnen mit Harnwegsinfektionen führen zu Blasenreizung, schmerzhaftem und häufigem Wasserlassen, Albuminurie und Hämaturie. Es gibt keine Hinweise auf eine systemische Wirkung von Hexamethylentetramin nach oraler Gabe bei Nagetieren.

Eine geringe mutagene Wirkung in bakteriellen Tests wird in vitro bei sehr hohen, jedoch nicht zytotoxischen Konzentrationen beobachtet. In vivo tritt keine Klastogenität in Chromosomenaberrationstests und einem Dominant-Letal-Test nach einmaliger oraler Gabe an Mäuse auf.

Hexamethylentetramin-Verabreichungen im Trinkwasser von bis zu 2000 mg/kg KG und Tag bis zu 104 Wochen lang führen bei Ratten und Mäusen nicht zur Tumorinduktion.

Es liegen keine bewertungsrelevanten Studien zur Entwicklungstoxizität vor.

## 2 Wirkungsmechanismus

Die Wirksamkeit von Hexamethylentetramin als antibakterielles Mittel bei Harnwegsinfektionen beruht auf seiner Fähigkeit, in saurer Umgebung langsam zu Formaldehyd und Ammoniak zu hydrolysieren (Dreyfors et al. 1989; Henschler 1991). Formaldehyd weist eine unspezifische antimikrobielle Aktivität auf, indem es Proteine und Nukleinsäuren in Bakterienzellen denaturiert. Die Wirksamkeit des Hexamethylentetramins ist abhängig von der Azidität des Urins (Li et al. 2024).

Formaldehyd wirkt kanzerogen an der Nase, wenn die Entgiftungskapazität des Nasengewebes überschritten ist (Greim 2000). Es gibt jedoch Überlegungen Hexamethylentetramin in der Tumorbehandlung einzusetzen, da der hypoxische Kern von Tumoren einen sauren pH-Wert aufweist und Hexamethylentetramin selektiv auf nicht proliferierende und behandlungsresistente Krebsklone in großen Tumoren (z. B. Glioblastom) wirken könnte. Das im sauren Milieu durch Hydrolyse entstandene Formaldehyd kann, wie in Bakterien, den Zelltod über Denaturierung von Proteinen und Nukleinsäuren auslösen (Altinoz et al. 2019).

Hexamethylentetramin verursacht selten Leberschäden, da es in der Leber nicht metabolisiert und schnell unverändert mit dem Urin ausgeschieden wird (ohne Verfasser:in 2021).

Aufgrund der chemischen Struktur werden keine oxidierenden Eigenschaften von Hexamethylentetramin angenommen (BAuA 2008). Die In-chemico-Untersuchung der Reaktion von Hexamethylentetramin in Phosphatpuffer bei pH 7,4 mit nukleophilen Aminosäuren ergab eine mögliche Reaktion mit Cystein, jedoch nicht mit Lysin. Dies könnte mit für die sensibilisierende Wirkung verantwortlich sein (Kireche et al. 2010).

Hexamethylentetramin war nur in zwei von 1044 durchgeführten In-vitro-Assays, die in der ToxCast-Datenbank gelistet sind, aktiv. Gemessen wurde eine verringerte *CYP1A1*- und *CYP1A2*-mRNA Expression in der humanen Leberzelllinie HepaRG, jeweils nur bei den beiden höchsten Konzentrationen (US EPA 2024).

## 3 Toxikokinetik und Metabolismus

Zur Bewertung der Formaldehydfreisetzung sind die Halbwertszeiten unter den Bedingungen an der Nasenschleimhaut mit einem pH-Wert von 6,5–7,0 wichtig (Kim et al. 2013).

Die Formaldehydfreisetzung von Hexamethylentetramin wurde bei pH-Werten von 2,0–7,4 sechs Stunden lang gemessen. Eingesetzt wurden je 7,5 mg Hexamethylentetramin in 10 ml 0,2 M Phosphatpuffer (pH-Einstellung mit 0,1 M Citratpuffer) bei 37,5 °C. Die Hydrolyseraten sowie die Halbwertszeiten sind in Tabelle 1 dargestellt (ECHA 2024; Henschler 1991; Strom und Jun 1980).

**Tab. 1:** Halbwertszeit-Bestimmung von Hexamethylentetramin bei verschiedenen pH-Werten und 37,5 °C (ECHA 2024; Strom und Jun 1980)

**Hydrolyse**	**pH-Wert**							
	**2,0**	**2,4**	**3,4**	**4,6**	**5,1**	** 5,5**	** 5,8**	**7,4**
Hydrolyserate pro Stunde	0,43	0,33	0,22	0,19	0,08	0,06	0,05	nicht messbar
Halbwertszeit [h]	1,6	2,12	3,09	3,73	8,29	10,9	13,8	–

h: Stunde

Eine Formaldehydabspaltung aus ^13^C-Hexamethylentetramin in Phosphatpuffer mit einem pH-Wert von 7,4 wurde 40 Tage lang mit ^13^C-NMR-Spektroskopie überprüft. Es wurde kein Formaldehyd freigesetzt (Kireche et al. 2010).

Die Formaldehydfreisetzung aus 750 µg Hexamethylentetramin/ml wurde in Urin („pooled“ Urin von drei Probanden) bei pH 4,9–6,5 und 37,5 °C untersucht. Der pH-Wert im Urin wurde mit 3 N Salzsäure eingestellt. Eine bakterizide Konzentration von > 28 µg Formaldehyd/ml wurde nach 1,5 Stunden bei pH 5,6, nach drei Stunden bei pH 5,8 und nach mehr als fünf Stunden bei pH 6 erreicht (Strom und Jun 1993). Aus der Graphik ist zu entnehmen, dass bei pH 6,5 nach sechs Stunden weniger als 10 µg Formaldehyd/ml aus den eingesetzten 750 µg/ml entstehen. 750 µg Hexamethylentetramin entsprechen 964 µg Formaldehyd. In sechs Stunden wird also ca. 1 % des Hexamethylentetramins hydrolysiert und damit in einer Stunde weniger als 0,2 %. In dieser Studie wurden deutlich geringere Geschwindigkeitskonstanten erhalten als von Strom und Jun (1980).

In einer weiteren Studie wurden in synthetischem Urin aus 1 mg Hexamethylentetramin/ml bei pH 6,5 ca. 10 µg Formaldehyd/ml freigesetzt (Musher und Griffith 1974).

### 3.1 Aufnahme, Verteilung, Ausscheidung

#### 3.1.1 Inhalative Aufnahme

Hierzu liegen keine Daten vor.

#### 3.1.2 Orale Aufnahme

Eine orale Verabreichung von 1 oder 2 g Hexamethylentetraminhippurat führte bei Patienten zu ca. 80%iger Aufnahme mit einem Maximum in Serum und Plasma nach ein bis zwei Stunden. Die Halbwertszeit im Plasma betrug vier Stunden. Im Blut liegt Hexamethylentetramin nicht hydrolysiert vor. Im Magen werden 10–30 % in Formaldehyd umgewandelt. Im Harn wird aufgrund der sauren Bedingungen Formaldehyd freigesetzt. Hexamethylentetramin passiert die Plazentaschranke. Eine genaue Darstellung der Daten erfolgte bei Henschler (1991). Nur die bei der Berechnung einer Luftkonzentration aus Studien mit oraler Verabreichung benötigte prozentuale Aufnahme bei Ratte und Mensch wird hier ausführlicher dargestellt.

Nach wiederholter zweimal täglicher oraler Verabreichung von 1 g Hexamethylentetraminhippurat an je sechs Probanden wurde nach zwölf Stunden ca. 80 % der Menge mit dem Urin ausgeschieden. Dies wurde über einen Zeitraum von sechs Tagen beobachtet (Allgén et al. 1979).

Je drei männlichen Sprague-Dawley-Ratten wurden 0,5 mg Hexamethylentetramin/kg KG in destilliertem Wasser per Schlundsonde verabreicht. Nach 0, 5, 10, 15, 30 und 45 Minuten sowie 1, 2, 3, 4, 6, 8, 10, 12 und 24 Stunden später erfolgte die Bestimmung von Hexamethylentetramin im Blutplasma. Es wurde nach 5–15 Minuten im Plasma nachgewiesen und erreichte nach 10–45 Minuten die maximale Konzentration. Die Halbwertszeit wurde auf 1,12 ± 0,19 Stunden geschätzt und die Bioverfügbarkeit betrug 89,83 % (Kim et al. 2023).

#### 3.1.3 Dermale Aufnahme

In einem Test nach OECD-Prüfrichtlinie 427 wurde männlichen Sprague-Dawley-Ratten je 5 mg von Acrylsäuregelen mit einem Gehalt von 0,15 % bzw. 0,6 % Hexamethylentetramin auf eine rasierte Hautfläche von 20,25 cm^2^ aufgetragen und für 24 Stunden okklusiv exponiert. Aus den Ergebnissen der engmaschig angelegten Plasmauntersuchungen nach Applikation wurde eine Bioverfügbarkeit von 78,91 % bzw. 77,19 % berechnet. Damit lässt sich eine perkutane Aufnahmegeschwindigkeit von etwa 0,25 µg/cm^2^/h bzw. 1 µg/cm^2^/h berechnen (Kim et al. 2023). Unter Standardbedingungen (2000 cm^2^ Hautfläche, eine Stunde Kontaktdauer) resultiert demnach eine Gesamtaufnahme von 0,5 mg bzw. 2 mg Hexamethylentetramin.

Nach Modellrechnungen mit IH SkinPerm v2.04 (Tibaldi et al. 2014) und Fiserova-Bergerova et al. (1990) werden aus einer gesättigten Lösung unter Standardbedingungen (eine Stunde Expositionsdauer, 2000 cm^2^ Hautfläche) zwischen 94 mg (0,67 mmol) und 467 mg (3,3 mmol) Hexamethylentetramin perkutan aufgenommen. Unter (stark) sauren Bedingungen können aus diesen Stoffmengen theoretisch 4 mmol (120 mg) bzw. 19,8 mmol (594 mg) Formaldehyd freigesetzt werden. Bei den pH-Werten der Haut (ca. 5,5) und im Blut (7,4) ist die Hydrolyse sehr langsam bzw. nicht mehr messbar (Tabelle 1). In experimentellen Untersuchungen mit Hexamethylentetramin in Urin bzw. Citratpuffer von Strom und Jun (1980, 1993) bei pH-Werten von 5,6 bzw. 5,8 betrug der Anteil des hydrolysierten Hexamethylentetramins etwa 5 %. Demnach können aus den berechneten perkutanen Aufnahmemengen theoretisch bis zu etwa 6 mg bzw. 30 mg Formaldehyd freigesetzt werden.

### 3.2 Metabolismus

Hexamethylentetramin hydrolysiert im Körper unter sauren Bedingungen zu Formaldehyd und Ammoniak. Das entstehende Formaldehyd reagiert direkt innerhalb einer Minute weiter zu Ameisensäure. Ameisensäure hat eine Halbwertszeit von 55 Minuten (BAuA 2008).

Die folgende Reaktionsgleichung zeigt die Hydrolyse im sauren Milieu:

(N_4_CH_2_)_6_ + 6 H_2_O + 4 H^+^ → 4 NH_4_^+^ + 6 HCHO

(Lo et al. 2014).

## 4 Erfahrungen beim Menschen

Hexamethylentetramin ist geruchlos (Dreyfors et al. 1989).

### 4.1 Einmalige Exposition

Hierzu liegen keine Daten vor.

### 4.2 Wiederholte Exposition

In einem Hexamethylentetramin produzierenden Betrieb wurde eine Querschnittstudie mit 33 Beschäftigten durchgeführt. Die Gruppe wurde unterteilt in 17 Personen (Baggerfahrer, Schichtleiter, leitende Angestellte, mittlere Dauer der Beschäftigung 115 Monate (79–152 Monate)), die als deutlich exponiert eingeschätzt wurden und 16 Beschäftigte als Kontrollgruppe, die nicht oder nur gelegentlich der Substanz ausgesetzt waren (mittlere Dauer der Beschäftigung 99 Monate (74–125 Monate)). Für jeden Beschäftigten wurden Anamnesedaten aufgezeichnet und eine Untersuchung der Haut und der Lunge durchgeführt. Darüber hinaus wurde das Gesamt- und spezifische IgE gegen vier Umweltallergene bestimmt, die Lungenfunktion und bronchiale Hyperreaktivität wurden beurteilt und es wurden Haut- und Epikutantests mit bekannten sensibilisierenden Substanzen sowie mit Hexamethylentetramin durchgeführt. Die geometrischen Mittelwerte personenbezogener Messungen der Hexamethylentetramin-Konzentrationen betrugen 0,6 mg/m^3^ (95-%-Konfidenzintervall (KI) 0,3–1,1) für Baggerfahrer und 0,3 mg/m^3^ (95-%-KI 0,1–0,9) für Schichtleiter. Ebenfalls bestimmt wurde die personenbezogene Konzentration an einatembarem Staub mit geometrischen Mittelwerten von 1,5 mg/m^3^ (95-%-KI 0,8–2,6) bzw. 0,7 mg/m^3^ (95-%-KI 0,2–2,0). Ortsfeste Messungen des einatembaren Staubes ergaben 1,1 mg/m^3^ (95-%-KI 0,6–2,0) an der Baggerstation und 0,7 mg/m^3^ (95-%-KI 0,4–1,2) in der Produktion und im Lager. Die Konzentration des alveolengängigen Staubes wurde nur an der Baggerstation mit 0,2; 0,3 und 0,7 mg/m^3^ bestimmt; er enthielt ca. 50 % Hexamethylentetramin. Zusätzlich wurden 1,2 mg Ammoniak/m^3^ und 0,22 mg Formaldehyd/m^3^ gemessen. Bei den spirometrischen Untersuchungen (FEV_1_ und MEF50 (maximale Ausatmungsgeschwindigkeit bei 50 % der forcierten Vitalkapazität)) wurden etwas schlechtere Ergebnisse bei den Kontrollen beobachtet. Außer einer verstärkten Sensibilisierung (Abschnitt 4.4) traten keine weiteren Unterschiede zwischen den beiden Gruppen auf (Merget et al. 1999).

Eine Studie mit Beschäftigten einer Reifenproduktionsanlage, die unter anderem gegen Hexamethylentetramin-Resorcin-Mischungen (2–3 % der Gesamtgummimasse) exponiert waren, ergab einen statistisch signifikant reduzierten exspiratorischen Fluss bei niedrigem Lungenvolumen und eine Verringerung der Ventilationskapazität. Jedoch konnten die Symptome nicht kausal dem Hexamethylentetramin zugeordnet werden (BAuA 2008; Gamble et al. 1976). Genaue Expositionsangaben fehlen.

Die Wirkung von Hexamethylentetramin auf wiederkehrende Harnwegsinfektionen wurde vergleichend mit Antibiotika-Gabe an Frauen (Alter > 18 Jahre) untersucht. Dazu erhielten 120 Patientinnen zwölf Monate lang eine tägliche orale Gabe von 2 g Hexamethylentetraminhippurat/Tag (je 1 g in einem zwölfstündigen Abstand) verabreicht. Hexamethylentetramin war ähnlich wirksam wie Antibiotika (50/100 mg Nitrofurantoin, 100 mg Trimethoprim oder 250 mg Cefalexin). Von den während der Studie aufgetretenen adversen Reaktionen wurden von den Autoren keine auf die Einnahme von Hexamethylentetramin zurückgeführt (Harding et al. 2022). Die häufigsten Nebenwirkungen, die in der Hexamethylentetramin-Gruppe berichtet wurden, waren Infektionen der unteren Atemwege (9 %), Bauchschmerzen (9 %) und Kopfschmerzen (7 %). Patientinnen, die Antibiotika erhielten, zeigten ähnliche Nebenwirkungen (Li et al. 2024).

Eine vergleichende Studie zur Behandlung wiederkehrender Harnwegsinfektionen wurde mit 45 Patientinnen, die 2 g Hexamethylentetraminhippurat/Tag (je 1 g in einem zwölfstündigen Abstand) 272,1 ± 18,4 Tage lang erhielten und mit 47 Patientinnen, die das Antibiotikum Trimethoprim erhielten, durchgeführt. Hexamethylentetraminhippurat war ähnlich wirksam wie das Antibiotikum. Patientinnen, die Hexamethylentetramin erhielten, berichteten von Durchfall (zwei Fälle), Bauchschmerzen (ein Fall) und Nierensteinen (ein Fall). Die Autoren vermuten keinen Zusammenhang mit der Hexamethylentetramingabe (Botros et al. 2022).

Für eine bakterizide Wirkung des Hexamethylentetramins in der Anwendung beim Menschen sollte eine Formaldehyd-Konzentration von 25 µg/ml Urin erreicht werden (Lo et al. 2014).

Die therapeutische orale Gabe von 8 g Hexamethylentetramin pro Tag führte bei < 3,5 % der Patienten zu adversen Effekten, die neben Kopfschmerzen, Atemnot, Magen-Darm-Beschwerden hauptsächlich Blasenreizung, schmerzhaftes und häufiges Wasserlassen, Albuminurie und Hämaturie umfassten. Bei Gabe von niedrigeren täglichen Dosen (2–4 g/Tag) wurden keine Nebenwirkungen beobachtet (BAuA 2008, ohne Verfasser:in 2021).

Eine Aufnahme von 57 mg/kg KG und Tag (4000 mg pro Tag bei einem durchschnittlichen Körpergewicht von 70 kg) wird von dem Scientific Committee on Health and Environmental Risks (SCHER) als NOAEL angesehen (SCHER 2007).

### 4.3 Wirkung auf Haut und Schleimhäute

In einem Betrieb, der zur Katalyse eines Herstellungsprozesses von Phenol-Formaldehyd-Harzen Hexamethylentetramin einsetzte, klagten fünf von 23 Beschäftigten über Reizwirkungen in der Nase und sechs über Augenreizungen. Es wurde nur die Konzentration von Formaldehyd bestimmt (im Atembereich), die unter der Nachweisgrenze der gewählten Methode von 1 ml/m^3^ lag (Low und Mitchell 1985). Aufgrund der fehlenden Angaben zur Hexamethylenkonzentration in der Luft kann die Studie nicht zur Bewertung herangezogen werden.

### 4.4 Allergene Wirkung

#### 4.4.1 Hautsensibilisierende Wirkung

Seit dem Nachtrag aus dem Jahr 1997 (Greim 1997) wurden verschiedene Untersuchungen veröffentlicht, die zeigen, dass Hexamethylentetramin hautsensibilisierend wirkt. Aktuell wird Hexamethylentetramin als 1%ige Testzubereitung in Vaseline als Bestandteil der „Gummireihe“ der Deutschen Kontaktallergie-Gruppe (DKG) getestet. International erfolgen Testungen auch mit einer 2%igen Testzubereitung in Vaseline.

##### 4.4.1.1 Studien ohne Unterteilung in beruflich und nicht-beruflich bedingte Kontaktdermatitis 

In zwei Studien mit Personen mit und ohne beruflich bedingter Kontaktdermatitis lag die Reaktionsquote jeweils bei 0,3 % (Zusammenfassung der Studien in Tabelle 2): In einer Auswertung des Informationsverbunds Dermatologischer Kliniken (IVDK) wurden zwischen 2007 und 2016 insgesamt 27 549 Personen mit Hexamethylentetramin (1 % in Vaseline) getestet, davon reagierten 83 (0,3 %) positiv (Geier und Schubert 2021). In einer anderen Studie reagierten drei von 931 (0,3 %) getesteten Patienten positiv auf Hexamethylentetramin (2 % in Vaseline) (Latorre et al. 2011).

##### 4.4.1.2 Studien mit Personen mit beruflicher Kontaktdermatitis

Während Hexamethylentetramin etwa zwischen 1970–1990 ein häufiges Allergen in der Gummiindustrie war, liegen aktuell nur wenige Fälle beruflicher allergischer Kontaktdermatitis mit belegter klinischer Relevanz vor (de Groot et al. 2010; de Groot und Flyvholm 2020). Positive Epikutantestreaktionen wurden für einzelne Beschäftigungsfelder beschrieben (z. B. Maschinentechnik, Massage, Frisörsalon, Lackiererei, Gärtnerei, Kosmetikinstitut (Aalto-Korte et al. 2008)). In Untersuchungen an größeren Kollektiven von Personen mit beruflich bedingter Kontaktdermatitis (zum Beispiel Metallarbeiter, Pflegepersonal, Beschäftigte im Umgang mit Epoxidharzen) liegen die Reaktionsquoten zwischen 0 und 2,2 % (Zusammenfassung der Studien in Tabelle 2).

In einer Auswertung des IVDK reagierten zwischen 2007 und 2016 50 (0,4 %) von 11 555 Personen mit beruflich bedingter Kontaktdermatitis positiv auf Hexamethylentetramin (1 % in Vaseline) (Geier und Schubert 2021).

Höhere Reaktionsquoten wurden in einer Studie gefunden, bei der die Testung mit der 2%igen Testzubereitung (in Vaseline) durchgeführt wurde, hierbei reagierten von getesteten 1495 Personen mit beruflich bedingter Kontaktdermatitis 26 (1,7 %) positiv (Aalto-Korte und Pesonen 2021).

Von 174 mit 2 % Hexamethylentetramin bei Verdacht auf eine Allergie gegen Kunst- und Klebstoffe getesteten Personen, die meisten davon mit beruflichem Zusammenhang, reagierten vier (2,2 %) positiv und eine Person zeigte irritative Reaktionen (0,6 %) (Kanerva et al. 1997).

Aus einem Gesamtkollektiv von 360 konsekutiv getesteten im Pflegebereich Beschäftigten mit beruflich bedingter Kontaktdermatitis wurden 72 mit Hexamethylentetramin (1 % in Vaseline) getestet, davon reagierten drei positiv (Nettis et al. 2002).

Im Zusammenhang mit Kühlschmierstoffen reagierte keiner der 71 getesteten Metallarbeiter auf die 1%ige Hexamethylentetramin-Testzubereitung (Geier et al. 2006).

In einer Untersuchung zu Kontaktallergien gegen Epoxidharze reagierte von insgesamt 585 getesteten Personen keine von 164 mit einer 2%igen Testzubereitung getesteten Personen, jedoch 7 (1,7 %) von 421 mit der 1%igen Testkonzentration getesteten. Die Reaktionen wurden jedoch als klinisch nicht relevant bewertet (Aalto-Korte et al. 2014).

Insgesamt 15 von 96 Personen mit beruflich bedingter Kontaktdermatitis reagierten auf 2 % Hexamethylentetramin (Aalto-Korte et al. 2008).

In einer Untersuchung zu Effekten von Hexamethylentetramin bei Beschäftigten einer Hexamethylentetramin-produzierenden Anlage wurden 17 Exponierte und 16 Kontrollpersonen aus demselben Betrieb untersucht. Des Weiteren wurden vier ehemalige Mitarbeiter untersucht, die in den letzten zehn Jahren aus medizinischen Gründen die Produktion (unklar ob Anlage oder Betrieb) verlassen hatten. Zwei der 17 Exponierten und zwei der 16 Kontrollpersonen berichteten über Dermatitis innerhalb des letzten Jahres, wobei die Symptome jedoch nur bei einer aktuell exponierten Person im beruflichen Zusammenhang standen und bei der zweiten Person bereits vor der Exposition bestanden. Epikutantests (1 % und 2 %, jeweils in Vaseline sowie wässrig) verliefen negativ, wobei mit anderen Allergenen positive Ergebnisse erhalten wurden. Unter den ehemals Beschäftigten, die die Produktion aus gesundheitlichen Gründen verlassen hatten, zeigten zwei ehemalige Baggerfahrer positive Ergebnisse im Epikutantest und berichteten über Ekzeme während der Exposition, welche aber nach Beendigung der Exposition abklangen (Merget et al. 1999).

##### 4.4.1.3 Studien mit Personen mit nicht-beruflicher Kontaktdermatitis

In zwei Studien, in denen Personen mit nicht-beruflicher Kontaktdermatitis auf eine Sensibilisierung gegen Hexamethylentetramin getestet wurden, lagen die Reaktionsquoten zwischen 0,2 und 0,45 % (Zusammenfassung der Studien in Tabelle 2): Von 12 359 Personen reagierten 24 (0,2 %) auf die 1%ige Testzubereitung (Geier und Schubert 2021). In einer weiteren Studie zu Kontaktallergien im Zusammenhang mit Kosmetika wurden europaweit 6050 Personen mit der gleichen Testzubereitung getestet, davon reagierten 27 (0,45 %) positiv. Mit der 2%igen Testzubereitung wurden in derselben Studie drei von 805 (0,37 %) Personen positiv getestet (Horton et al. 2021).

##### 4.4.1.4 Studien mit Personen ohne bekannte Kontaktallergie

In einer Untersuchung zu Kontaktsensibilisierungen im Rahmen einer Kosmetikserie reagierten drei (1,5 %) von 201 getesteten Freiwilligen ohne bekannte Kontaktallergie auf Hexamethylentetramin (2 % in Vaseline) (Zhao und Li 2014). In einer nachfolgenden Untersuchung desselben Zentrums reagierten drei (0,6 %) von 481 Freiwilligen auf dieselbe Testzubereitung (Zhao und Li 2015).

##### 4.4.1.5 Studien mit gleichzeitiger Testung auf Formaldehyd

Einige Studien beschäftigen sich auch mit dem Zusammenhang zwischen Reaktionen auf Hexamethylentetramin und Formaldehyd. Von Personen, die positiv auf Hexamethylentetramin reagierten, reagierte in verschiedenen Untersuchungen auch die überwiegende Mehrheit auf Formaldehyd:

Von 26 positiv auf Hexamethylentetramin (2 % in Vaseline) getesteten Personen reagierten 22 auch auf Formaldehyd (1 % wässrig) (Aalto-Korte und Pesonen 2021). In zwei weiteren Studien derselben Gruppe reagierten von insgesamt 15 Personen, die positiv auf Hexamethylentetramin reagierten, auch neun auf Formaldehyd (Aalto-Korte et al. 2008) bzw. sechs von sieben Personen auch auf Formaldehyd (Aalto-Korte et al. 2014). In einer weiteren Studie reagierten von drei positiv auf Hexamethylentetramin (2 % in Vaseline) getesteten Personen zwei auch positiv auf Formaldehyd (1 % wässrig) (Latorre et al. 2011).

Umgekehrt reagierten von 78 positiv auf Formaldehyd getesteten Patienten neun auch auf Hexamethylentetramin (2 % in Vaseline) (Aalto-Korte et al. 2008). In einer größeren Studie reagierten von 402 positiv auf Formaldehyd getesteten Personen 26 (6,5 %) auch positiv auf Hexamethylentetramin (1 % in Vaseline), während von 25 077 negativ auf Formaldehyd (1 % wässrig) reagierenden Personen 44 (0,2 %) positiv auf Hexamethylentetramin reagierten (Geier 2022).

Aufgrund der, allerdings nur langsamen, Hydrolyse von Hexamethylentetramin unter Freisetzung von Formaldehyd im Sauren (s. Abschnitt 3) können die aufgetretenen positiven Epikutantestreaktionen auf Hexamethylentetramin als Reaktion auf Formaldehyd bewertet werden. Außerdem zeigen In-chemico-Untersuchungen, dass Hexamethylentetramin noch weitere Reaktionsprodukte mit Aminosäuren (insbesondere Cystein) bilden kann (Kireche et al. 2010).

##### 4.4.1.6 Einzelfälle

Bei einer Biochemikerin, die mit Polyacrylamid-Gelen umging, wurde zunächst eine einfach positive Reaktion beobachtet, bei Nachtestung jedoch eine als irritativ bewertete Reaktion auf Hexamethylentetramin (k. A. zur Testzubereitung) beobachtet (Aalto-Korte et al. 2002). In einem weiteren Fall wurde ein Lackierer mit beruflicher Epoxiddermatitis mit positiven Epikutantest-Reaktionen auf mehrere Härter negativ auf 2 % Hexamethylentetramin getestet (Kanerva et al. 1997).

Zwischen 1996 und 2012 wurden fünf Fälle von Hexamethylentetramin-Sensibilisierungen im beruflichen Zusammenhang mit Natur- und Synthesekautschukprodukten an ein Überwachungssystem im Vereinigten Königreich gemeldet (k. A. zur Testzubereitung und Anzahl der insgesamt getesteten Personen) (Warburton et al. 2015).

Einzelfälle positiver Epikutantestreaktionen im nicht-beruflichen Kontext wurden im Zusammenhang mit einem Hexamethylentetramin-haltigen Antitranspirant (González-Pérez et al. 2003) und mit einem in Schienbeinschonern enthaltenen Epoxidhärter berichtet (Sommer et al. 1999). In beiden Fällen reagierten die Patienten auch positiv auf Formaldehyd. Ein weiterer Einzelfall einer positiven Reaktion im Kontext mit vermuteter Allergie gegen Gummiinhaltsstoffe trat bei einem Kind auf (Nettis et al. 2001).

**Tab. 2:** Studien zu Hautreaktionen auf Hexamethylentetramin in Epikutantests bei Patienten mit Verdacht auf Kontaktallergie ab 1995

**getestete Personen**	**Konzentration, in Vaseline**	**Ergebnis:** **Reaktion bei**	**Bemerkungen^a)^**	**Literatur**
**Studien mit Personen ohne Unterteilung in berufliche und nicht-berufliche Kontaktdermatitis**				
27 549	1 %	83 von 27 549 (0,4 %)	Testzeitraum: 2007–2016; Kollektiv: IVDK-Studie, Gesamtkollektiv; Ablesezeitpunkt und Ergebnis: D3, in Ausnahmefällen D4	Geier und Schubert 2021
931	2 %	3 von 931 (0,3 %)	Testzeitraum: 2005–2009; Kollektiv: konsekutiv getestete Personen (609 ♀, 322 ♂, 62 % ≥ 40 Jahre alt) im Rahmen einer Multizenterstudie; Ablesezeitpunkt und Ergebnis: D2 und D4; 2 der positiv reagierenden Personen auch positiv auf Formaldehyd	Latorre et al. 2011
**Studien mit Personen mit beruflicher Kontaktdermatitis**				
11 555	1 %	50 von 11 555 (0,4 %)	Testzeitraum: 2007–2016; Kollektiv: IVDK-Studie, Personen (k. w. A.) mit beruflich bedingter Kontaktdermatitis, keine Aufschlüsselung nach Berufsgruppen; Ablesezeitpunkt und Ergebnis: D3, in Ausnahmefällen D4	Geier und Schubert 2021
1495	2 %	26 von 1495 (1,7 %)	Testzeitraum: 2007–2020; Kollektiv: konsekutiv getestete Personen (k. w. A.) mit beruflich bedingter Kontaktdermatitis, Studie zu Formaldehydabspaltern; Ablesezeitpunkt und Ergebnis: D2, D3 und D4 oder D6 bzw. D2 und D5; 22 der positiv reagierenden Personen reagierten auch gleichzeitig auf Formaldehyd	Aalto-Korte und Pesonen 2021
585	1 % 2 %	7 von 421 (1,7 %) 0 von 164 (0 %)	Testzeitraum: 1991–2013; Kollektiv: Aus einem Gesamtkollektiv mit 688 Personen (k. w. A.) mit beruflicher Kontaktdermatitis im Zusammenhang mit Epoxidharzen wurden 585 getestet; Ablesezeitpunkt und Ergebnis: D2, D3 und D4 oder D6 bzw. D2 und D5, positive Reaktionen wurden jedoch als klinisch nicht relevant bewertet; zusätzlich: mit beiden Testzubereitungen jeweils 1 × Irritation	Aalto-Korte et al. 2014
174	2 %	4 von 174 (2,2 %)	Testzeitraum: 3 Jahre (k. w. A.); Kollektiv: Personen (k. w. A.) mit allergischer oder irritativer Kontaktdermatitis und Verdacht auf eine Allergie gegen Kunst- und Klebstoffe, die meisten davon im beruflichen Kontext; Ablesezeitpunkt und Ergebnis: D2, D3 und D4–6; zzgl. 1 Irritation (0,6 %)	Kanerva et al. 1997
71	1 %	0 von 71	Testzeitraum: 2000–2002 und 2004–2005; Kollektiv: Von insgesamt 144 Metallarbeitern mit beruflich bedingter irritativer oder allergischer Kontaktdermatitis (29 ♀, 135 ♂, Alter: 19–62 Jahre) wurden 71 getestet; Ablesezeitpunkt und Ergebnis: D3, in Ausnahmefällen D4	Geier et al. 2006
72	1 %	3 von 72	Testzeitraum: 1994–1998; Kollektiv: Von 360 konsekutiv getesteten im Pflegebereich Beschäftigten mit beruflich bedingter Kontaktdermatitis (280 ♀, 80 ♂, Alter zwischen 22 und 74 Jahren) wurden 72 getestet; Ablesezeitpunkt und Ergebnis: D2 und D4	Nettis et al. 2002
37	1 % 2 % 1 % wässrig 2 % wässrig	0 von 17 aktuell Exponierten, 0 von 16 Kontrollpersonen, 2 von 4 ehemals Exponierten	Testzeitraum: k. A.; Kollektiv: Querschnittsstudie, 37 ♂, 2/17 und 2/16 mit irritativer oder allergischer Kontaktdermatitis innerhalb des letzten Jahres, aktuelle Symptome nur bei 1/17 aktuell Exponierten, 2 ehemals Exponierte reagierten positiv und berichteten über Ekzeme während der Exposition, welche nach Beendigung der Exposition abklangen; Ablesezeitpunkt und Ergebnis: D2 und D3. K. A., mit welcher Testzubereitung die positiven Ergebnisse beobachtet wurden	Merget et al. 1999
96	2 %	15 von 96, davon: 9 von 78 mit Formaldehyd-Allergie, 6 von 18 ohne Formaldehyd-Allergie	Testzeitraum: 2001–2007; Kollektiv: Aus einem Gesamtkollektiv von Personen (k. w. A.) mit beruflich bedingter Kontaktdermatitis, davon 81 mit Allergie gegen Formaldehyd und 18 ohne Allergie gegen Formaldehyd, wurden 78 + 18 getestet; Ablesezeitpunkt und Ergebnis: D2, teilweise D3, und D4, 5 oder 6; Angaben zur Reaktionsstärke nur zu Personen ohne Formaldehyd-Allergie: 6 × 2+ Reaktion	Aalto-Korte et al. 2008
**Studien mit Personen mit nicht beruflich-bedingter Kontaktdermatitis**				
12 359	1 %	24 von 12 359 (0,2 %)	Testzeitraum: 2007–2016; Kollektiv: IVDK-Studie, Personen mit nicht-beruflich bedingter Kontaktdermatitis; Ablesezeitpunkt und Ergebnis: D3, in Ausnahmefällen D4	Geier und Schubert 2021
6050 805	1 % 2 %	27 von 6050 (0,45 %) 3 von 805 (0,37 %)	Testzeitraum: 2009–2018; Kollektiv: europaweite ESSCA Multizentrum-Studie, konsekutiv getestete Personen mit Kontaktallergien zum Zusammenhang mit Kosmetika (k. w. A.); Ablesezeitpunkt und Ergebnis: k. A zum Ablesezeitpunkt (vermutlich D2 und D3 oder D4); 22 der positiv reagierenden Personen reagierten auch gleichzeitig auf Formaldehyd	Horton et al. 2021
**Studien mit Personen ohne bekannte Kontaktallergie**				
201	2 %	3 von 211 (1,5 %)	Testzeitraum: 2011; Kollektiv: Personen ohne bekannte Kontaktallergie (140 ♀, 61 ♂, Alter: 19–30, durchschnittlich 23 Jahre alt); Ablesezeitpunkt und Ergebnis: D2 und D4	Zhao und Li 2014
481	2 %	3 von 481 (0,6 %)	Testzeitraum: 2014; Kollektiv: Personen ohne bekannte Kontaktallergie (332 ♀, 149 ♂, Alter: 18–33, durchschnittlich 24 Jahre alt); Ablesezeitpunkt und Ergebnis: D2 und D4	Zhao und Li 2015

^a)^ keine Angabe über zeitlichen Reaktionsverlauf, keine Angabe über Reaktionsstärken, sofern nicht angegeben

D: Tag nach Applikation; ESSCA: European Surveillance System on Contact Allergies; IVDK: Informationsverbund Dermatologischer Kliniken; k. A.: keine Angabe; k. w. A: keine weiteren Angaben; zzgl.: zuzüglich

##### 4.4.1.7 Fazit

Neue klinische Befunde zeigen weiterhin eine kontaktallergene Wirkung von Hexamethylentetramin.

#### 4.4.2 Atemwegssensibilisierende Wirkung

In einer Untersuchung zu Effekten von Hexamethylentetramin auf die Atemwege wurden 17 Beschäftigte einer Hexamethylentetramin-produzierenden Anlage untersucht, die gegen mittlere geometrische Hexamethylentetramin-Konzentrationen zwischen 0,3 und 0,6 mg/m^3^ exponiert waren. Weiterhin wurden 16 Kontrollpersonen aus demselben Betrieb sowie vier ehemalige Mitarbeiter untersucht, die in den letzten zehn Jahren aus medizinischen Gründen die Produktion (unklar ob Anlage oder Betrieb) verlassen hatten. Elf der 17 exponierten Personen berichteten über Symptome (Kurzatmigkeit, Husten, brennende oder juckende Augen, Rhinitis, Dermatitis) während des letzten Jahres, wobei die Symptome jedoch nur bei zwei Personen im Zusammenhang mit der beruflichen Exposition standen. Von den 16 Kontrollpersonen berichteten elf über Symptome, wobei nur in einem Fall ein Arbeitsplatzbezug angegeben wurde. Bezüglich Gesamt- und spezifischem Immunglobin E gegen vier Umweltallergene, Lungenfunktion und bronchialer Reaktionsfähigkeit auf Methacholin, gab es keine Unterschiede zwischen den Gruppen. Hautpricktests (100 mg/ml) und Epikutantests (1 % und 2 %, jeweils in Vaseline sowie wässrig) verliefen negativ, wobei mit anderen Umweltallergenen positive Ergebnisse erhalten wurden. Insgesamt war bei den vorliegenden Expositionskonzentrationen an Hexamethylentetramin keine Evidenz für ein erhöhtes Risiko für beruflich bedingtes allergisches Asthma zu beobachten (Merget et al. 1999).

### 4.5 Reproduktionstoxizität

Aus Untersuchungen an Frauen, die während der Schwangerschaft mit Hexamethylentetramin als antibakteriell-antiseptisches Arzneimittel gegen Harnwegsinfektionen behandelt worden waren, haben sich keine substanzbedingten Auffälligkeiten im Schwangerschaftsverlauf oder in der Entwicklung der Kinder ergeben. Die Mütter erhielten therapeutische Dosen von 2 g Hexamethylentetraminhippurat pro Tag (ca. 0,9 g Hexamethylentetramin) oder 4 g Hexamethylentetraminmandelat pro Tag (ca. 1,9 g Hexamethylentetramin). Dies entspricht etwa 13 bis 27 mg Hexamethylentetramin/kg KG und Tag, berechnet auf der Grundlage eines angenommenen Körpergewichts von 70 kg (BAuA 2008).

### 4.6 Genotoxizität

Hierzu liegen keine Daten vor.

### 4.7 Kanzerogenität

In Bezug auf die umfangreiche Verwendung von Hexamethylentetramin als Arzneimittel liegen keine Informationen über die Bildung von Tumoren im Harntrakt beim Menschen vor (BAuA 2008).

#### 4.7.1 Fall-Kontroll-Studien

Hierzu liegen keine Daten vor.

#### 4.7.2 Kohortenstudien

Eine Kohortenstudie mit 13 570 männlichen Personen, die im Zeitraum von 1940–1976 mindestens fünf Jahre in einer Gummiproduktions-Firma beschäftigt waren, wurde durchgeführt. Dabei bestand eine Exposition gegen Hexamethylentetramin und zahlreiche weitere Substanzen, bei denen zum Teil ein Verdacht auf eine kanzerogene Wirkung vorlag. Es wurden die Mortalitäts- und Krebsmorbiditätsraten nach Arbeitsbereich im Vergleich mit einer standardisierten Rate männlicher US-Amerikaner ermittelt. Positive Assoziationen (beobachtete Fälle zu erwarteten Fällen) wurden für alle Tumortypen ermittelt. Diese konnten jedoch nicht einzelnen Substanzen zugeordnet werden (BAuA 2008).

Auch in einer weiteren Kohorte mit 12 743 Personen konnte kein kausaler Zusammenhang zwischen Lungenkrebs und Hexamethylentetramin-Exposition hergestellt werden, da Co-Expositionen gegen Ammoniak, Antioxidantien, Asbest, Ruß, Farbstoffe/Pigmente, Melamin, Partikel, Phenol, Weichmacher, Harnstoffverbindungen und Holzstaub vorlagen (BAuA 2008).

Erhöhte Raten an Blasenkrebs wurden in einer Kohortenstudie mit 632 Formenbauern in einer Gießerei im Vergleich mit anderen weniger exponierten Facharbeitern beobachtet. Auch hier lag neben der Exposition gegen Hexamethylentetramin eine Co-Exposition u. a. gegen promovierende Substanzen wie Phenole, Kresole und Aldehyde vor. Ein kausaler Zusammenhang mit einer Hexamethylentetramin-Exposition konnte daher nicht abgeleitet werden (BAuA 2008).

## 5 Tierexperimentelle Befunde und In-vitro-Untersuchungen

### 5.1 Akute Toxizität

#### 5.1.1 Inhalative Aufnahme

Je fünf männliche und weibliche Wistar-Ratten wurden nach OECD-Prüfrichtlinie 403 vier Stunden nur über die Nase gegen 0; 1,81; 3,34 oder 5,28 g Kofasil-Liquid (16,5 % Hexamethylentetramin und 25,5 % Natriumnitrit)/m^3^ exponiert. Die Aerosole hatten einen massenmedianen aerodynamischen Durchmesser von 2,55; 2,67 bzw. 3,21 µm. Keine der Ratten der niedrigsten Konzentrationsgruppe verendete, aber die Sterblichkeit betrug 70 % bzw. 90 % in den Gruppen mit mittlerer und hoher Konzentration. Bei den verendeten Tieren wurden vergrößerte Herzen und Flüssigkeit in der Lunge festgestellt. Einige der überlebenden Tiere, darunter auch diejenigen, die gegen die niedrigste Konzentration exponiert waren, wiesen ebenfalls vergrößerte Herzen und eine Erweiterung der Herzkranzgefäße auf. Die 4-Stunden-LC_50_ von Kofasil-Liquid bei Wistar-Ratten wurde mit 3,15 (2,44–3,89) g/m^3^ berechnet (k. w. A.; EFSA FEEDAP Panel 2015). Eine weitere Quelle gibt als Inhaltsstoffe des Handelsproduktes Kofasil Ultra Hexamethylentetramin, Natriumnitrit, Natriumbenzoat und Natriumpropionat an. Die Dichte betrug 1,16–1,18 g/cm^3^ und der pH-Wert ca. 8,0–9,5. Kofasil wird beim Silierprozess verwendet (ADDCON GmbH 2024).

#### 5.1.2 Orale Aufnahme

Für die Maus wurden orale LD_50_-Werte von 1853 bis > 5000 mg/kg KG angegeben; Intoxikationssymptome wurden nicht berichtet (Henschler 1991).

#### 5.1.3 Dermale Aufnahme

Hierzu liegen keine Daten vor.

### 5.2 Subakute, subchronische und chronische Toxizität

#### 5.2.1 Inhalative Aufnahme

Es liegen keine Inhalationsstudien mit Hexamethylentetramin vor.

#### 5.2.2 Orale Aufnahme

Je 15 weibliche und männliche Sprague-Dawley-Ratten erhielten 50 Wochen lang an fünf Tagen in der Woche 0 oder 0,1 % Hexamethylentetramin im Trinkwasser. Die Tiere wurden bis zum Ende ihrer Lebenszeit weiter gehalten und dann erst wurde bei allen Tieren eine Nekropsie und die komplette histopathologische Untersuchung durchgeführt. Es wurden keine Effekte beobachtet (ECHA 2024; Henschler 1991; Lijinsky und Taylor 1977). Die Konzentrationen im Trinkwasser (0,1 % entspricht 1000 mg/l) betrugen 0 oder ca. 50 mg/kg KG (Umrechnungsfaktor 0,05 (chronisch) nach EFSA Scientific Committee (2012)). Aus dieser Studie kann ein NOAEL von 50 mg/kg KG abgeleitet werden.

In einer 60-wöchigen Studie erhielten 50–99 männliche und 50–120 weibliche CTM-Mäuse 0; 0,5 oder 1,0 % Hexamethylentetramin im Trinkwasser. Zusätzlich wurde 29 männlichen und 50 weiblichen CTM-Mäusen 30 Wochen lang 5,0 % Hexamethylentetramin im Trinkwasser verabreicht. Nur bei den Tieren der hohen Dosis wurde eine höhere Sterblichkeit nachgewiesen. Bei den Tieren mit geringerer Hexamethylentetramin-Verabreichung entsprach die Körpergewichtsentwicklung und die Sterblichkeit den Kontrollwerten. Die mikroskopische Untersuchung ergab bei allen Tieren keine Effekte (Della Porta et al. 1968; ECHA 2024; Henschler 1991). Die Hexamethylentetramin-Konzentrationen im Trinkwasser (0,5 % entspricht 5000 mg/l, 1 % entspricht 10 000 mg/l, 5 % entspricht 50 000 mg/l) betrugen ca. 250, ca. 500 bzw. ca. 2500 mg/kg KG (Umrechnungsfaktor 0,05 (chronisch) nach EFSA Scientific Committee (2012)).

Nach 90-tägiger Schlundsondengabe von 400 mg Hexamethylentetramin/Tier und Tag (ca. 820 mg/kg KG und Tag für männliche und 1450 mg/kg KG und Tag für weibliche Ratten) an je zehn weibliche und männliche Albino-Ratten wurden keine Effekte beobachtet, mit Ausnahme einer Gelbverfärbung der Haare. Auch Schlundsondengaben über einen Zeitraum von 333 Tagen von 400 mg pro Tier und Tag an je 15 weibliche und männliche Ratten führten in Verhalten, Entwicklung und Gewichtszunahme nicht zu Unterschieden zu den Kontrolltieren, außer einer Gelbverfärbung der Haare (Brendel 1964; Henschler 1991).

Über die gesamte Lebensdauer mit dem Futter verabreichte Dosierungen von 0 oder 100 mg Hexamethylentetramin/kg KG und Tag im Futter bewirkten keine Effekte bei je 16 weiblichen und männlichen Wistar-Ratten. Untersucht wurde Muskelaktivität, Körpergewicht, relatives Organgewicht und Allgemeinzustand (Henschler 1991; Natvig et al. 1971).

In einer Kanzerogenitäts-Studie über einen Zeitraum von 104 Wochen erhielten je 48 männliche und weibliche Wistar-Ratten 0 oder 1 % Hexamethylentetramin im Trinkwasser. Die Körpergewichtsentwicklung und die Sterblichkeit entsprachen denen der Kontrolltiere. Die mikroskopische Untersuchung ergab keine Effekte (Della Porta et al. 1968; ECHA 2024; Henschler 1991). Die Konzentration im Trinkwasser entsprach ca. 500 mg/kg KG (Umrechnungsfaktor 0,05 (chronisch) nach EFSA Scientific Committee (2012)).

In einer Fütterungsstudie erhielten je zwei männliche und drei weibliche Katzen zwei Jahre lang ca. 60 mg Hexamethylentetramin/kg KG und Tag. Untersucht wurden Futterkonsum, Körpergewichtsentwicklung und Verhalten. Es traten keine Effekte auf (BAuA 2008; ECHA 2024, unveröffentlicht, Durchführung 1963–1966).

**Fazit**: Nach Verabreichung von 0,1 % Hexamethylentetramin im Trinkwasser über einen Zeitraum von 50 Wochen wurden bei der histopathologischen Untersuchung keine Effekte beobachtet. Die Konzentrationen im Trinkwasser betrugen 1000 mg/l (ca. 50 mg/kg KG, Umrechnungsfaktor 0,05 (chronisch) nach EFSA Scientific Committee (2012)). Damit stellt 50 mg/kg KG den NOAEL dar. Die mit „mikroskopischer Untersuchung“ bezeichneten Gewebe-Untersuchungen der älteren Studien werden als weniger valide angesehen, da davon ausgegangen wird, dass die durchgeführte Histopathologie in der vergleichsweise neueren Studie von Lijinsky und Taylor (1977) den wahrscheinlich höchsten Standard aufweist.

#### 5.2.3 Dermale Aufnahme

Eine nicht-okklusive dermale Auftragung von 2 ml einer 0,2%igen wässrigen Hexamethylentetramin-Lösung an fünf Tagen in der Woche sechs Wochen lang bewirkte bei je sechs Kaninchen keine Effekte auf Verhalten, Körpergewichtsentwicklung oder Haarwachstum. An der Haut wurden keine Effekte beobachtet (k. w. A.; ohne Verfasser:in 1992).

### 5.3 Wirkung auf Haut und Schleimhäute

#### 5.3.1 Haut

Bei drei Kaninchen (Weiße Russen, k. w. A.) löste eine okklusive Auftragung von 0,5 g Hexamethylentetramin, angefeuchtet mit 120 µl Wasser, im 4-Stunden-Patch-Test auf der intakten rasierten Haut keine Reizwirkungen aus (ECHA 2024; Henschler 1991).

Sechs männliche Kaninchen (k. A. zur Rasse) wurden mit einer 0,2%igen wässrigen Hexamethylentetramin-Lösung auf intakter bzw. abradierter Flankenhaut 24 Stunden okklusiv behandelt. Die Tiere wurden 72 Stunden beobachtet und zeigten eine leichte Reizwirkung (k. w. A.; ohne Verfasser:in 1992).

Je sechs männlichen Kaninchen (k. A. zur Rasse) wurde Wasser oder eine 0,2%ige wässrige Hexamethylentetramin-Lösung an fünf Tagen in der Woche sechs Wochen lang aufgetragen. Die Exposition erfolgte nicht-okklusiv. Es wurden keine Effekte beobachtet (k. w. A.; ohne Verfasser:in 1992).

Hexamethylentetramin ist nach dem global harmonisierten System (GHS) von den REACH-Registranten nicht als hautreizend eingestuft (ECHA 2024).

Nach OECD-Prüfrichtlinie 404 wurde bei weißen Neuseeländer-Kaninchen Kofasil-Liquid (16,5 % Hexamethylentetramin und 25,5 % Natriumnitrit) auf Hautreizung getestet. Die Exposition der Haut führte zu Erythemen, Ödemen und leichten Narbenbildungen. Die Erytheme waren noch 72 Stunden nach der Exposition sichtbar (k. w. A.; EFSA FEEDAP Panel 2015). Eine weitere Quelle gibt als Inhaltsstoffe des Handelsproduktes Kofasil Ultra Hexamethylentetramin, Natriumnitrit, Natriumbenzoat und Natriumpropionat an. Die Dichte betrug 1,16–1,18 g/cm^3^ und der pH-Wert ca. 8,0–9,5 (ADDCON GmbH 2024). Weder Natriumnitrit, Natriumbenzoat noch Natriumpropionat zeigen eine reizende Wirkung an der Haut (ECHA 2025 b, c, d).

**Fazit**: Nach vierstündiger okklusiver Auftragung war angeteigtes Hexamethylentetramin nicht hautreizend beim Kaninchen.

#### 5.3.2 Auge

Die Instillation von 0,1 g in den Konjunktivalsack des Auges löste bei je drei männlichen und weiblichen Kaninchen (k. A. zur Rasse) keine Reizwirkungen nach Draize aus. Bei allen drei Tieren trat eine Stunde nach der Anwendung eine leicht verstärkte Sekretion auf, die innerhalb von 24 Stunden reversibel war. Die Augen wurden nicht gespült (k. w. A.; BAuA 2008; ECHA 2024; Henschler 1991).

Die Instillation von 0,1 ml einer wässrigen 0,2%igen Hexamethylentetramin-Lösung in den Konjunktivalsack von sechs männlichen Kaninchen (k. A. zur Rasse) führte nicht zu Reizwirkungen. Die Augen wurden nicht gespült (k. w. A.; ohne Verfasser:in 1992).

Hexamethylentetramin ist nach dem global harmonisierten System (GHS) von den REACH-Registranten nicht als augenreizend eingestuft (ECHA 2024).

**Fazit**: Hexamethylentetramin ist nicht augenreizend.

### 5.4 Allergene Wirkung

#### 5.4.1 Hautsensibilisierende Wirkung

Neben dem bereits in der letzten Begründung aufgeführten positiven Maximierungstest liegen nun ebenfalls positive Ergebnisse aus einem unvollständig dokumentierten Local Lymph Node Assay an weiblichen BALB/c-Mäusen vor. Es wurden vier Konzentrationen zwischen 2,5 und 20 % in Aceton/Olivenöl (4:1 V/V) getestet. Im ersten Test wurden die Tiere nach Prüfrichtlinie zu Versuchsbeginn, am 1. und 2. Tag behandelt und nach fünf Tagen die Proliferation der Lymphknotenzellen bestimmt. Als Stimulationsindex wird 4,0 angegeben. In einem weiteren Test wurden die Tiere zu Versuchsbeginn, am 1. und 2. Tag sowie sieben Wochen lang wöchentlich behandelt. Die Proliferation der Lymphknotenzellen wurde am 61. Tag bestimmt, wobei ein Stimulationsindex von 5,8 angegeben wird (ECHA 2024). Insgesamt ist das Ergebnis als positiv zu bewerten, es fehlen jedoch Angaben zur Anzahl der Tiere pro Dosisgruppe. Ferner ist unklar, bei welcher getesteten Konzentration die Stimulationsindices bestimmt wurden, sodass das Ergebnis nur begrenzt aussagekräftig ist.

Das Ergebnis im Prädiktionsmodell OECD QSAR v4.7 war positiv (OECD 2024). Weitere Untersuchungen in nicht-tierbasierten Alternativverfahren (NAMs) liegen nicht vor, sodass kein Gesamtergebnis aus NAMs ermittelt werden kann.

#### 5.4.2 Atemwegssensibilisierende Wirkung

Es liegen keine Untersuchungen vor.

### 5.5 Reproduktionstoxizität

#### 5.5.1 Fertilität

In der Begründung von 1991 (Henschler 1991) werden mehrere Studien an Ratten mit Gabe vor und während der Verpaarung aufgeführt, bei denen es bis zur höchsten verabreichten Dosis von ca. 100 mg/kg KG und Tag über das Futter nicht zu Effekten auf die Fertilität kam.

#### 5.5.2 Entwicklungstoxizität

Seit der Begründung von 1991 (Henschler 1991) sind keine neuen Daten veröffentlicht worden.

In der Begründung von 1991 wird ein modifizierter Chernoff-Kavlock-Test zum Nachweis potentieller Teratogene aufgeführt. In diesem Test wurden neun Alpk:AP-Ratten vom 7. bis zum 17. Gestationstag mit 1000 mg Hexamethylentetramin/kg KG und Tag per Schlundsonde behandelt. Die Muttertiere wiesen eine verzögerte Körpergewichtszunahme auf. Effekte auf Wurfgröße, Überlebensrate und Gewichtszunahme der Nachkommen bis zum 5. Postnataltag wurden nicht festgestellt. Hexamethylentetramin wurde in diesem Test nicht als Teratogen bewertet. Eine spezifische Untersuchung der Teratogenität wird bei diesem Test nicht vorgenommen (Henschler 1991; Wickramaratne 1987).

Zudem wird in der Begründung von 1991 eine Studie an Beagle-Hündinnen beschrieben. In dieser Studie wurden je acht bis neun Tieren vom 4. bis zum 56. Tag nach der Paarung (gesamte Gestationsdauer 66 Tage) 0, 600 oder 1250 mg Hexamethylentetramin/kg Futter (0, 15 bzw. 31 mg/kg KG und Tag) verabreicht. Behandlungsbedingte Effekte auf Trächtigkeitsrate, Körpergewichtszunahme der trächtigen Tiere, Gestationsdauer und Wurfgröße wurden nicht festgestellt. Bei 31 mg/kg KG und Tag war die Anzahl der Totgeburten leicht erhöht (bedingt durch einen Wurf mit nur zwei lebenden von insgesamt neun Welpen) und die Gewichtszunahme sowie Überlebensrate der Welpen bis zum Absetzen leicht erniedrigt. Organ- und Skelettfehlbildungen wurden nicht festgestellt (Henschler 1991; Hurni und Ohder 1973).

### 5.6 Genotoxizität

#### 5.6.1 In vitro

Der Test auf differenzielle Abtötung mit Bacillus subtilis („Rec-Assay“) mit 1000 µg Hexamethylentetramin/Platte ergab ein positives Ergebnis in Bacillus subtilis H17 und M45 (Henschler 1991). Die Publikation liegt mit Ausnahme des Abstracts und der Tabellen auf Japanisch vor.

Ein Test auf differenzielle Abtötung mit E. coli („polA-Assay“) verlief positiv bei 6000 µg/Platte, jedoch nicht bei 500 µg/Platte (Henschler 1991).

Im Green Screen Assay (DNA-Reparatur Reporter-Assay mit Saccharomyces cerevisiae) zeigte Hexamethylentetramin positive Effekte ohne metabolische Aktivierung ab 150 µg/ml (Cahill et al. 2004). Das Testsystem ist nicht validiert.

Mutagenitätstests mit den Salmonella-typhimurium-Stämmen TA97, TA98, TA100, TA1535, TA1537 und TA1538 wurden mit und ohne Zusatz eines metabolischen Aktivierungssystems im Bereich 100–10 000 µg/Platte in zwei verschiedenen Laboren durchgeführt. Nur TA97 und TA100 zeigten reproduzierbar erhöhte Revertantenzahlen bei beginnender Zytotoxizität und wurden als fraglich mutagen bewertet. Eine Statistik wurde nicht angegeben (Zeiger et al. 1992). Es wurden keine Verdoppelungen der Revertantenzahlen beobachtet. Die Erhöhung wurde nur bei sehr hohen Konzentrationen festgestellt (abgeschätzt: 6000 und 10 000 µg/Platte), was die vorgeschriebene höchste Dosierung von 5000 µg/Platte (OECD-Prüfrichtlinie 471) übersteigt.

Mutagenitätstests mit den Salmonella-Stämmen TA98 und TA100 zeigten mit und ohne Zusatz eines metabolischen Aktivierungssystems keine Mutagenität (k. w. A.). Als Kriterium für ein positives bzw. fraglich positives Ergebnis wurde hier eine 2- bzw. 1,7-fache Erhöhung der Revertantenzahl festgelegt (Takahshi und Ono 1993).

Mit den Salmonella-Stämmen TA98, TA100, TA1535, TA1537, TA1538 und E. coli WP2uvrA wurden Mutagenitätstests mit Konzentrationen von 5–10 000 µg Hexamethylentetramin/Platte mit und ohne Zusatz eines metabolischen Aktivierungssystems durchgeführt. Nur in den Stämmen TA98 und TA100 wurde eine zweifache Erhöhung der Revertanten bei sehr hohen Konzentrationen von 10 000 µg/Platte und damit Mutagenität beobachtet. Eine Statistik wurde nicht angegeben (Shimizu et al. 1985).

In den Salmonella-Stämmen TA98 und TA100 traten nach Inkubation mit 200–5000 µg Hexamethylentetramin/Platte keine Mutationen im Mutagenitätstest mit und ohne Zusatz eines metabolischen Aktivierungssystems auf (Henschler 1991).

Bei einer Konzentration von 1000 µg Hexamethylentetramin/Platte wurde im Salmonella-Mutagenitätstest in den Stämmen TA98, TA100, TA1535, TA1537 und TA1538 keine Mutagenität nachgewiesen (Henschler 1991).

Ein weiterer Mutagenitätstest fand keine Mutagenität durch Hexamethylentetramin in den Stämmen TA98, TA100, TA1535, TA1537 und TA1538 (k. w. A.; Henschler 1991). Die eingesetzten Konzentrationen pro Platte sind nicht angegeben.

Die Inkubation von V79-Zellen mit 10 oder 50 mmol Hexamethylentetramin/l bewirkte leicht erhöhte Schwesterchromatidaustausch-Häufigkeiten, die statistisch signifikant waren (BAuA 2008; Girmanová et al. 1991).

In HeLa-Zellen wurden erhöhte Chromosomenaberrationen ohne metabolische Aktivierung bei einer Konzentration von 1 mM Hexamethylentetramin beobachtet. Zytotoxizität trat bei 14 mM auf. In humanen Leukozyten wurden keine Chromatid- oder Chromosomenaberrationen beobachtet (k. w. A.; Henschler 1991). Da für die HeLa-Zelllinie hohe chromosomale Instabilität beschrieben ist (Frattini et al. 2016), wird dieser Test nicht zur Bewertung herangezogen.

In einem Chromosomenaberrationstest mit V79-Zellen führte die höchste auswertbare Konzentration von 10 mmol/l (ca. 1,4 mg/ml) zu 7 % veränderten Zellen im Vergleich mit den Kontrollzellen, die nur 2 % veränderte Zellen aufwiesen. Die Veränderung war nicht statistisch signifikant und damit ist das Ergebnis negativ. Höhere Konzentrationen waren stark zytotoxisch (Girmanová et al. 1991). Für eine genaue Beurteilung wurden in dieser Studie zu wenige Metaphasen ausgewertet.

Ein TK^+/–^-Test, der negativ verlief, wurde an Mauslymphomzellen durchgeführt (Henschler 1991). Die Daten liegen nur als Konferenzabstract vor. Eine Konzentration ist nicht angegeben.

#### 5.6.2 In vivo

In zwei Chromosomenaberrationstests mit einmaliger oder fünftägiger oraler Verabreichung (69–618 mg/kg KG) wurde keine Genotoxizität im Knochenmark von männlichen C3H-Mäusen beobachtet (BAuA 2008; Henschler 1991).

Im Dominant-Letaltest wurde nach einmaliger oraler Verabreichung der sehr hohen Dosis von 25 000 mg/kg KG an männliche C3H-Mäuse eine statistisch signifikante Erhöhung toter Implantate, jedoch nicht eine Vermehrung präimplantativer Eiverluste beobachtet. Diese Dosis wurde von den Tieren nach oraler Gabe „gerade noch vertragen“ und war, intraperitoneal verabreicht, letal. Die einmalige intraperitoneale Gabe von 8000 oder 10 000 mg/kg KG an die männlichen Mäuse führte zu negativen Ergebnissen im Dominant-Letaltest (Henschler 1991). Der Dominant-Letaltest mit oraler Gabe wird wegen der hohen Dosierung nicht für die Bewertung herangezogen.

#### 5.6.3 Fazit

Hexamethylentetramin ist in bakteriellen Mutagenitätstests bei extrem hohen Konzentrationen schwach positiv. In V79-Zellen traten leicht erhöhte Schwesterchromatidaustausch-Häufigkeiten auf. In vivo wird in zwei Chromosomenaberrationstests mit oraler Gabe im Knochenmark von Mäusen und einem Dominant-Letaltest mit intraperitonealer Gabe keine Klastogenität beobachtet. Für die Mutagenität liegen keine In-vivo-Untersuchungen vor.

### 5.7 Kanzerogenität

#### 5.7.1 Kurzzeitstudien

Im Zelltransformationstest mit BHK-21/cl.13-Zellen und bei Zusatz eines metabolischen Aktivierungssystems (S9-Mix aus Lebern von mit Aroclor 1254 behandelten Ratten) bewirkte Hexamethylentetramin im Konzentrationsbereich von 1 bis 10 000 µg/ml einen dosisabhängigen Anstieg der Transformationsrate. Der toxische und transformierende Effekt bei 1000 µg Hexamethylentetramin/ml war ähnlich wie der von 20 µg Formaldehyd/ml (Henschler 1991). Das Testsystem ist nicht validiert und die Methode unzureichend dokumentiert (BAuA 2008).

#### 5.7.2 Langzeitstudien

Nach Hexamethylentetramin-Gabe im Trinkwasser 50, 60 oder 104 Wochen lang, sowie in Fütterungsstudien und Studien mit subkutaner Gabe über die gesamte Lebensdauer trat bei Ratten und Mäusen keine erhöhte Kanzerogenität auf. In Trinkwasser-Studien erhielten männliche und weibliche Wistar-Ratten 0 oder 1 % (ca. 500 mg/kg KG und Tag) über einen Zeitraum von 104 Wochen und Sprague-Dawley-Ratten 50 mg/kg KG und Tag an fünf Tagen pro Woche über einen Zeitraum von 50 Wochen. In der 60-wöchigen Trinkwasserstudie erhielten männliche und weibliche CTM-Mäuse 0, 250 oder 500 mg Hexamethylentetramin/kg KG und Tag (Abschnitt 5.2.2; ECHA 2024; Henschler 1991). Auch weitere bei Henschler (1991) beschriebene Studien zur Kanzerogenität von Hexamethylentetramin lassen keine kanzerogene Wirkung beim Menschen erwarten. Es liegen keine neuen Daten vor. Die in Prüfrichtlinien für Kanzerogenitätstests vorgegebenen Tierzahlen pro Dosisgruppe wurden nicht in allen Studien erreicht.

## 6 Bewertung

Das grundlegende Vorgehen zur Bewertung eines Arbeitsstoffes ist der MAK- und BAT-Werte-Liste zu entnehmen (DFG 2025).

Kritischer Effekt ist die hautsensibilisierende Wirkung bei Mensch und Tier (Greim 1997; Hartwig 2010).

**MAK-Wert. **Es liegen keine Humandaten oder Inhalationsstudien am Tier vor, aus denen ein MAK-Wert abgeleitet werden kann.

Eine Hydrolyse von Hexamethylentetramin zu Formaldehyd ist bei den physiologischen pH-Werten des Atemtrakts nur in einem sehr geringen Umfang zu erwarten (Abschnitt 3).

Der Dampfdruck von Hexamethylentetramin von etwa 0,0013 hPa (ECHA 2024) zeigt, dass die Substanz als Dampf und Aerosol vorliegen kann. Erst bei einem Dampfdruck von weniger als 10^–5^ hPa liegen Substanzen in der Luft weitgehend als Aerosol vor (DFG 2025). Die Dampfsättigungskonzentration von Hexamethylentetramin beträgt 1,3 ml/m^3^ (7,5 mg/m^3^).

Das Auftreffen von Aerosolen kann zu einer höheren (punktuellen) lokalen Konzentration in den Atemwegen führen (Aerosol-Impaktierung). Aufgrund der sehr guten Löslichkeit von Hexamethylentetramin ist aber von einer vollständigen Lösung im Lungen-Surfactant (pH 6,9; Amirkhanian und Merritt 1995) und im Mukus und einer Diffusion weg vom Auftreffort auszugehen, sodass lokal höhere Konzentrationen im Lungengewebe damit nicht wahrscheinlich sind.

Die Partikelgröße für ein kommerzielles Produkt beträgt 500 bis 3350 µm für 80 % aller Partikel. Es wurden keine Partikel unter 125 µm gefunden (ECHA 2024). Damit sind die Partikel nicht alveolengängig. Ob dies für alle Produkte zutrifft, ist nicht bekannt.

Die vorliegenden Daten belegen eine sehr geringe Reizwirkung an der Haut, jedoch keine Augenreizung. Damit ist auch keine Reizwirkung im Atemtrakt zu erwarten.

Zur Ermittlung des NOAEL wird die Studie mit dem wahrscheinlich höchsten Standard der histopathologischen Untersuchung herangezogen. Nach Verabreichung von Trinkwasser mit 0,1 % Hexamethylentetramin an Ratten (ca. 50 mg/kg KG) über einen Zeitraum von 50 Wochen wurden bei der histopathologischen Untersuchung keine Effekte beobachtet (Lijinsky und Taylor 1977, Abschnitt 5.2). Damit stellt 50 mg/kg KG den NOAEL dar.

Zur toxikokinetischen Übertragung dieses NOAEL von 50 mg/kg KG in eine Konzentration in der Luft am Arbeitsplatz werden berücksichtigt: der dem toxikokinetischen Unterschied zwischen Ratte und dem Menschen entsprechende speziesspezifische Korrekturwert (1:4), die angenommene orale Resorption der Ratte (90 %, Kim et al. 2023), das Körpergewicht (70 kg) und das Atemvolumen (10 m^3^) des Menschen, die angenommene 100%ige inhalative Resorption und die Übertragung der Daten aus dem Tierversuch auf den Menschen (1:2). Damit errechnet sich eine entsprechende Konzentration von 39,4 mg/m^3^. Mit dem Preferred Value Approach ergibt sich ein MAK-Wert von 20 mg/m^3^** ≙ **3,4 ml/m^3^ (ppm) für die E-Fraktion.

Bei Patienten treten keine adversen Effekte bei einer therapeutischen Gabe von 2–4 g Hexamethylentetraminhippurat/Tag auf. Zum Vergleich mit den Tierdaten wird eine toxikokinetische Übertragung dieser Dosis in eine Konzentration in der Luft am Arbeitsplatz vorgenommen. Dafür werden berücksichtigt: die angenommene orale Resorption beim Menschen (80 %, Allgén et al. 1979) und das Atemvolumen (10 m^3^) am Arbeitsplatz sowie die angenommene 100%ige inhalative Resorption. Damit errechnet sich eine entsprechende Konzentration von 160–320 mg Hexamethylentetraminhippurat/m^3^, das entspricht ca. 70–140 mg Hexamethylentetramin/m^3^.

**Krebserzeugende Wirkung. **Es gibt keine Daten, die auf eine krebserzeugende Wirkung von Hexamethylentetramin hinweisen. Die vorliegenden Tests zur genotoxischen Wirkung in vitro zeigen eine geringe mutagene Wirkung in bakteriellen Tests bei sehr hohen, jedoch nicht zytotoxischen Konzentrationen. In-vivo-Chromosomenaberrationstests mit oraler Gabe an der Maus sind negativ. Auch ein Dominant-Letal-Test mit einmaliger intraperitonealer Gabe an Mäuse zeigt keine Induktion von Letalmutationen.

Die lokale Kanzerogenität von Formaldehyd ist ausführlich dokumentiert (Greim 2000). Formaldehyd ist in Kanzerogenitäts-Kategorie 4 eingestuft, da es bei Konzentrationen, die die Entgiftungskapazitäten des Nasengewebes überschreiten, dort kanzerogen wirkt.

Eine Hydrolyse von Hexamethylentetramin in der nasalen Mucosa mit einem pH-Wert von 6,5 des Mukus (Kim et al. 2013) ist nur in einem sehr geringen Umfang zu erwarten, da in der Hydrolyse-Studie (Abschnitt 3) bei einem pH-Wert von 7,4 keine Hydrolyse stattfand und bei einem pH-Wert von 5,8 eine Halbwertszeit von 13,6 Stunden gemessen wurde. Auch die Messungen im Urin belegen, dass bei einem pH-Wert von 6,5 nur eine sehr geringe Hydrolyse stattfindet. Von den eingesetzten 750 µg Hexamethylentetramin/ml Urin wurden nur weniger als 0,02 %/h umgesetzt (Strom und Jun 1993).

Bei Exposition in Höhe des MAK-Wertes von 20 mg Hexamethylentetramin/m^3^ würde es bei einem achtstündigen Atemvolumen von 10 m^3^ höchstens zu einer Aufnahme von 25 mg pro Stunde kommen. Die Hydrolyserate bei einem pH-Wert von 5,8 beträgt 5 % pro Stunde, also wird ca. 1,25 mg Hexamethylentetramin hydrolysiert, was 1,6 mg Formaldehyd entspricht. Da aber in der Nase ein pH-Wert von 6,5–7,0 vorliegt, würde bei einem MAK-Wert von 20 mg Hexamethylentetramin/m^3^ weniger als 1,6 mg Formaldehyd pro Stunde freigesetzt.

Es werden folgende theoretische Überlegungen zur Prüfung der Entgiftungskapazität des entstehenden Formaldehyds aus Hexamethylentetramin vorgenommen: Formaldehyd wird schnell durch die Glutathion-abhängige Formaldehyddehydrogenase (Alkoholdehydrogenase 5) und *S*-Formylglutathionhydrolase zu Ameisensäure metabolisiert. Die geschätzte Halbwertszeit beträgt 1–1,5 Minuten in Humanzellen (EFSA 2014). Die intrazelluläre Konzentration von etwa 12 mg Formaldehyd/l (400 µM) wurde anhand von Messungen im Nasengewebe geschätzt (EFSA ANS Panel 2013). Der pH-Wert von Hexamethylentetramin liegt bei 8,4 bei 28 g/l und der pKs-Wert bei 8,95. Das weitere Hydrolyseprodukt Ammoniak neutralisiert zusammen mit dem nicht hydrolysierten Hexamethylentetramin den Mukus und führt somit zu noch geringerer Hydrolyse in der nasalen Mukosa. Es ist daher nicht anzunehmen, dass durch die geringe Hydrolyse von Hexamethylentetramin und damit nur geringe Freisetzung von Formaldehyd die Entgiftungskapazität des Nasengewebes überschritten wird.

Aufgrund der sehr guten Löslichkeit von Hexamethylentetramin sind hohe lokale Konzentrationen durch eine Impaktierung des Lungengewebes nicht wahrscheinlich (siehe „MAK-Wert“).

Basierend auf der sehr geringen Hydrolyse bei physiologischen pH-Werten im Atemtrakt und der Abschätzung, dass die Formaldehyd-Entgiftungskapazitäten des Nasengewebes nicht überschritten werden, wird Hexamethylentetramin nicht in eine Kanzerogenitäts-Kategorie eingestuft.

**Spitzenbegrenzung. **Da bei Hexamethylentetramin aufgrund der fehlenden Reizwirkung keine sofortige Wirkung im Vordergrund steht, wird es in Spitzenbegrenzungs-Kategorie II eingestuft. Die Halbwertszeit im Plasma beim Menschen ist vier Stunden. Daraus würde sich ein Überschreitungsfaktor von 4 ergeben (Hartwig 2011). Da aber eine 10-fach so hohe Dosis, die in anderen, wenn auch schlechter durchgeführten, Studien ohne Effekt blieb, wird ein Überschreitungsfaktor von 8 festgelegt.

**Fruchtschädigende Wirkung. **Aus der Anwendung als Arzneimittel beim Menschen liegen keine geeigneten Daten zur Bewertung der Entwicklungstoxizität vor.

Es gibt keine pränatalen Entwicklungstoxizitätsstudien, die nach gültigen Prüfrichtlinien durchgeführt worden sind. Aus einer Art Screening-Studie, einem modifizierten Chernoff-Kavlock-Test, an Alpk:AP-Ratten mit Schlundsondengabe von 1000 mg Hexamethylentetramin/kg KG und Tag vom 7. bis zum 17. Gestationstag (Greim 1997; Wickramaratne 1987) und einer Studie an trächtigen Beagle-Hündinnen mit Gabe von bis zu 31 mg Hexamethylentetramin/kg KG und Tag über das Futter vom 4. bis zum 56. Tag nach der Paarung (Greim 1997; Hurni und Ohder 1973) ergaben sich keine Hinweise auf eine entwicklungstoxische Wirkung.

Aufgrund des Fehlens belastbarer Daten zur pränatalen Entwicklungstoxizität wird Hexamethylentetramin der Schwangerschaftsgruppe D zugeordnet. Auf Basis der wenigen vorliegenden Daten lässt sich ein Verdacht auf eine entwicklungstoxische Wirkung (Gruppe B (Verdacht)) nicht begründen.

**Keimzellmutagene Wirkung. **Eine geringe mutagene Wirkung in bakteriellen Tests bei sehr hohen, jedoch nicht zytotoxischen Konzentrationen wurde in vitro beobachtet. In vivo verliefen Chromosomenaberrationstests im Knochenmark von Mäusen negativ. In Keimzellen der Maus wurden nach intraperitonealer Gabe keine Dominant-Letalmutationen induziert.

Aufgrund der sehr geringen Hydrolyse bei physiologischen pH-Werten ist nicht davon auszugehen, dass das freigesetzte Formaldehyd aus Hexamethylentetramin in aktiver Form die Keimzellen erreicht.

Hexamethylentetramin wird nicht in eine Kategorie für Keimzellmutagene eingestuft.

**Hautresorption. **Experimentelle Daten zur Hautresorbierbarkeit des Hexamethylentetramins wurden in einer tierexperimentellen Studie an Ratten ermittelt (Kim et al. 2023). Aus den Ergebnissen lässt sich für Standardbedingungen (2000 cm^2^ Hautfläche, eine Stunde Applikationsdauer) eine maximale Aufnahme von etwa 2 mg Hexamethylentetramin aus einem Gel mit 0,6 % Hexamethylentetramin berechnen. Modellrechnungen für Standardbedingungen und gesättigter Lösung lassen erwarten, dass zwischen 94 mg (Tibaldi et al. 2014) und 467 mg (Fiserova-Bergerova et al. 1990) Hexamethylentetramin resorbiert werden. Hexamethylentetramin ist weitgehend stabil im Blut. Bei der Worst-Case-Annahme, dass 5 % pro Stunde hydrolysiert und daraus Formaldehyd entsteht, werden maximal 30 mg Formaldehyd innerhalb einer Stunde freigesetzt (Abschnitt 3.1). Demnach werden maximal etwa 0,625 mg Formaldehyd/1,25 Minuten resorbiert bzw. freigesetzt (mittlere Halbwertszeit des Formaldehyds im Blut; Kaden et al. 2010). Der physiologische Formaldehydspiegel im Blut des Menschen beträgt etwa 2–3 mg/l (10–15 mg in 5 l Blut, frei und reversibel gebunden) (Heck et al. 1985). Die transdermale Aufnahme in den letzten sechs Halbwertszeit-Intervallen führt demnach im Fließgleichgewicht zu einer im Blut zirkulierenden Formaldehydmenge von (0,625 + 0,31 + 0,16 + 0,08 + 0,04 + 0,02) mg = 1,24 mg bei pH-Werten von 5,6 bzw. pH 5,8. Tatsächlich wird diese Menge geringer sein, da Blut einen pH-Wert von 7,4 aufweist. Diese Erhöhung der Formaldehyd-Gesamtmenge im Blut liegt im Variationsbereich der physiologischen Belastung, sodass Hexamethylentetramin nicht mit „H“ markiert wird.

**Sensibilisierende Wirkung. **Es liegen weiterhin klinische Befunde zur kontaktallergenen Wirkung von Hexamethylentetramin vor. Eine neue tierexperimentelle Untersuchung liefert ebenfalls einen Hinweis auf eine sensibilisierende Wirkung. Es liegen nicht ausreichend Einzeldaten vor, sodass kein Gesamturteil aus nicht tierbasierten Alternativverfahren (NAMs) ermittelt werden kann. In der Summe erfolgt, auch aufgrund der Eigenschaft von Hexamethylentetramin Formaldehyd freizusetzen, weiterhin eine Markierung mit „Sh“. Eine sensibilisierende Wirkung auf die Atemwege kann aus den vorliegenden Informationen nach wie vor nicht abgeleitet werden. Es erfolgt daher weiterhin keine Markierung mit „Sa“.
